# From colostomy creation to full enteral feeding in neonates with an anorectal malformation: evaluating the role of central venous access

**DOI:** 10.3389/fsurg.2025.1524404

**Published:** 2025-02-05

**Authors:** D. Huijgen, I. K. Schokker-van Linschoten, H. P. Versteegh, C. E. J. Sloots

**Affiliations:** Department of Pediatric Surgery, Erasmus University Medical Center—Sophia Children’s Hospital, Rotterdam, Netherlands

**Keywords:** anorectal malformation, neonatal surgery, colostomy, nutrition, central venous catheter, peripherally inserted central catheter

## Abstract

**Purpose:**

After creating a colostomy in newborns with anorectal malformations (ARMs), reaching full enteral feeding may take longer than expected, resulting in an unanticipated period of starvation. This retrospective cohort study aimed to evaluate the postoperative course regarding enteral feeding tolerance and the necessity for a central venous access device (CVAD) after colostomy formation in newborns with ARMs.

**Methods:**

The files of neonates with ARMs who underwent colostomy formation between January 2014 and August 2023 were reviewed. The primary outcome was the postoperative tolerance of enteral feeding. Secondary outcomes were the need for a CVAD and CVAD-related complications.

**Results:**

Thirty-four neonates with an ARM underwent colostomy formation. Enteral feeding was initiated on median postoperative day two (IQR 1–2). Full enteral feeding was reached on median postoperative day six (IQR 4–8). In nine neonates (26.5%), it took more than seven postoperative days to reach full enteral feeding, of whom seven (77.8%) had one or more comorbidities that could affect neonatal feeding tolerance. A CVAD was placed in 17 neonates (50%), of whom four (23.5%) needed additional general anesthesia for its placement. There were one or more CVAD-related complications in seven of 17 (41.2%) neonates, mainly involving suspicion of mild catheter-related infections.

**Conclusions:**

The majority of neonates undergoing colostomy formation for an ARM require more than five days to achieve full enteral feeding. It is recommended to bridge this period of inadequate feeding with either fluids or parenteral nutrition by inserting a CVAD during colostomy formation, particularly for those with comorbidities affecting neonatal feeding tolerance.

## 1 Introduction

Anorectal malformations (ARMs) are a spectrum of congenital malformations involving the distal rectum and anus. In patients with sufficient meconium discharge through either a perineal or vestibular fistula, initial treatment predominantly consists of dilatation of the fistula with subsequent elective reconstructive surgery. In patients without sufficient meconium discharge within the first 24 hours of life, surgical management encompasses colostomy creation with delayed definitive repair (1, 2).

Early enteral feeding following pediatric colorectal surgery is proven to be safe and leads to improved postoperative outcomes, such as a shorter length of stay and fewer postoperative complications (3–6). However, early initiation of enteral feeding may not always be feasible in neonates with an ARM who have undergone colostomy creation. Additionally, achieving full enteral feeding may take longer than expected. In a cohort study by Wolf et al., the time to initiation of enteral feeding after ostomy creation in neonates with various intestinal conditions ranged from one to 13 days, and the time to full enteral feeding from six to 209 days (7). However, for neonates undergoing colostomy formation for an ARM specifically, numbers on postoperative enteral feeding are lacking, resulting in an unforeseen period of insufficient enteral nutrition.

During this period of inadequate enteral nutrition, patients rely on intravenous (IV) fluid infusions or parenteral nutrition (PN). In cases of prolonged IV fluid or PN requirement, providing central venous access may be beneficial. However, since it is uncertain how long it takes to establish full enteral feeding after colostomy formation in neonates with an ARM, there exists some controversy in our center regarding whether central venous access is needed for these patients. Therefore, this retrospective cohort study aims to evaluate the postoperative course regarding enteral feeding tolerance and the subsequent need for central venous access after colostomy formation in neonates with an ARM.

## 2 Methods

### 2.1 Study design

A single-center retrospective observational cohort study was conducted at the Erasmus University Medical Center—Sophia Children's Hospital, Rotterdam, The Netherlands. All neonates with an ARM who underwent colostomy formation between January 2014 and August 2023 were included. Neonates who died before enteral feeding was initiated were excluded.

### 2.2 Data collection

The electronic patient files were reviewed to collect baseline characteristics (e.g., sex, gestational age, birth weight, 5-min Apgar score) and disease-specific parameters (e.g., ARM type, anomalies within the VACTERL association, other structural congenital anomalies, genetic syndromes). The daily fluid requirements in the first seven days of life were calculated based on age and birthweight according to hospital protocol (Table 1). The day of birth was determined as day zero; from day eight onward, 150 ml/kg was defined as full enteral feeding.

**Table 1:** Daily fluid requirements in the first 7 days after birth.

Birthweight	Day 1	Day 2	Day 3	Day 4	Day 5	Day 6	Day 7	
1–2 kg	60	80	100	120	140	160	180	ml/kg/day
>2 kg	20	60	80	120	140	150	150	ml/kg/day

The day of birth is considered to be day zero.

### 2.3 Outcomes

The primary outcome was the postoperative tolerance of enteral feeding, measured as the median number of postoperative days until both initiation of enteral feeding and reaching full enteral feeding. When assessing the primary outcome, groups were divided into patients with and without comorbidities that could potentially affect neonatal feeding tolerance [e.g., preterm birth, small for gestational age (<p10), major structural cardiac defect, tracheoesophageal anomaly, abdominal anomaly other than ARM, cerebral anomaly, syndromic condition]. Secondary outcomes were the need for a central venous access device (CVAD)—comprising non-tunneled central venous catheters (CVCs) and deep-seated peripherally inserted central catheters (PICCs)—and CVAD-related complications. Furthermore, the post-operative course (e.g., 30-day postoperative complication rate and duration of hospitalization) was evaluated. Complications were classified according to Clavien-Madadi (8).

### 2.4 Statistical analysis

Data was analyzed using SPSS (IBM SPSS Statistics for Windows, version 28.0, released in 2021, IBM Corp). No statistical tests were used due to the study's descriptive nature.

## 3 Results

### 3.1 Patient characteristics

During the study period, 153 ARM patients were treated in our institution, of whom 36 underwent colostomy formation in the neonatal phase. Two neonates were excluded due to death from multiple comorbidities before enteral feeding was initiated. Both of these neonates had a CVAD inserted, but no complications related to the CVAD occurred in either case. Finally, 34 neonates were included in the analysis. Table 2 shows an overview of the baseline characteristics, including comorbidities. Colostomy formation occurred one day after birth (IQR 1–2). The first colostomy output was observed on median postoperative day one (IQR 1–2). An overview of postoperative complications is shown in Table 3.

**Table 2:** Baseline characteristics.

	*n* = 34
Male sex, *n* (%)	29 (85.3)
Premature (<37 weeks of pregnancy), *n* (%)	3 (8.8)
Small for gestational age (birthweight <10th percentile), *n* (%)	13 (38.2)
5 min Apgar score, median (IQR)	9 (9–10)
Type or anorectal malformation, *n* (%)	
Perineal fistula^a^	3 (8.8)
Vestibular fistula^a^	1 (2.9)
Urethral fistula	19 (55.9)
Bladder neck fistula	3 (8.8)
No fistula	4 (11.8)
Cloacal malformation	4 (11.8)
Anomalies within VACTERL, *n* (%)	
Vertebral	6 (17.6)
Cardiac	21 (61.8)
Tracheoesophageal	5 (14.7)^b^
Renal	16 (47.1)
Limb	3 (8.8)
Other structural congenital anomalies, *n* (%)	
Gastroenterological (other than esophageal atresia) anomalies	2 (5.9)
Internal/external genital anomalies	7 (20.1)
Cerebral/spinal anomalies	5 (14.7)
Ear nose throat anomalies	3 (8.8)
Others	2 (5.9)
Genetic syndromes, *n* (%)	6 (17.6)

^a^ Colostomy formation was performed as dilation of the fistula was insufficient.

^b^ Two cases of esophageal atresia, two cases of tracheomalacia, and one case of a narrow trachea.

**Table 3:** Postoperative complications within 30 days.

	*n* = 34 *n* (%)	Clavien-Madadi classification
High output stoma	11 (32.4)	100% Grade IB
Suspicion of CVAD related- sepsis	6 (17.6)	66.7% Grade IB, 33.3% Grade II
Wound infection/dehiscence	4 (11.8)	75% Grade IB, 25% Grade II
Urosepsis	4 (11.8)	100% Grade II
CVAD dislodgement	2 (5.9)	50% Grade IB, 50% Grade IIIA
Fever without a clear focus	1 (2.9)	Grade II
Esophageal anastomotic leakage with pneumothorax	1 (2.9)	Grade IV
Catheter-induced urethral injury	1 (2.9)	Grade IB
Nasogastric tube-induced gastric perforation	1 (2.9)	Grade IV
Sagittal sinus thrombosis	1 (2.9)	Grade II
Atelectasis	1 (2.9)	Grade IB

### 3.2 Enteral feeding

**Figure 1:**
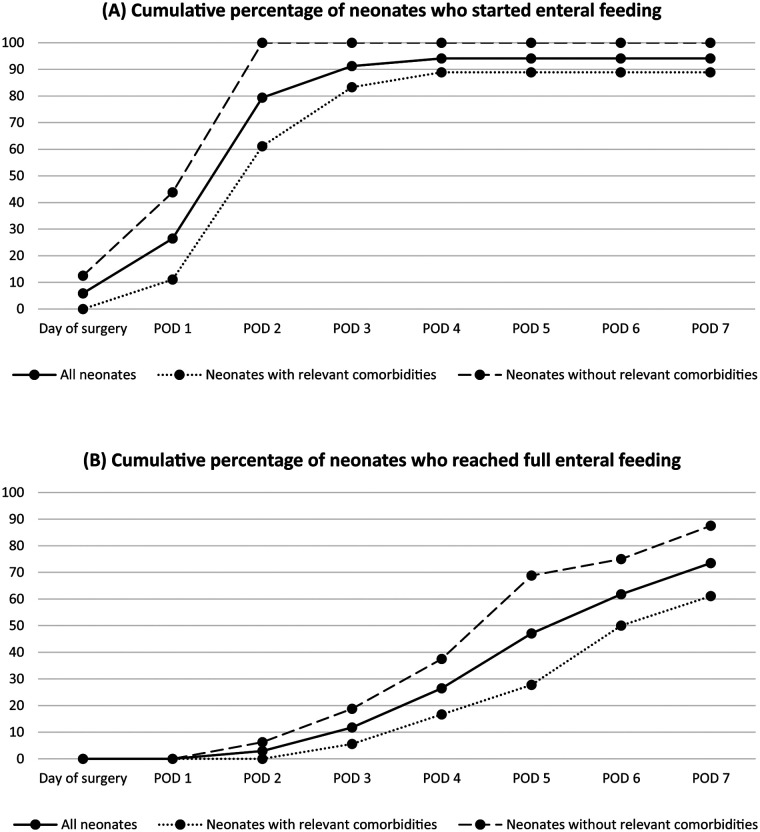
Postoperative course regarding enteral feeding. The cumulative percentages are presented for all neonates included in the study (*n* = 34), the neonates with comorbidities affecting neonatal feeding tolerance (*n* = 18), and the neonates without any of these comorbidities (*n* = 16). **(A)** Cumulative percentage of neonates who initiated enteral feeding in the first seven days after surgery **(B)** cumulative percentage of neonates who reached full enteral feeding in the first seven days after surgery.

Figure 1 shows the cumulative percentage of neonates for whom enteral feeding was initiated (Figure 1A) and who reached full enteral feeding (Figure 1B) within the first seven postoperative days. The percentages are presented for the entire study population and subgroups with and without comorbidities potentially affecting neonatal feeding tolerance. When considering all 34 neonates, enteral feeding was initiated on median postoperative day two (IQR 1–2). In 28 patients (82.4%), enteral feeding was initiated on the day of or one day after the first colostomy output. In two patients (5.9%), enteral feeding was initiated up to two days before the first colostomy output. In the remaining four patients (11.8%), enteral feeding could not be initiated at the time of the first colostomy output due to contraindications (prolonged postoperative ventilation in one, persistent bilious drainage from the nasogastric tube in two—of whom one also had duodenal atresia with annular pancreas—and esophageal anastomosis leakage in one). As a result, enteral feeding for these patients was initiated between two and 18 days after the first colostomy output. Among the 34 neonates, full enteral feeding was reached on median postoperative day six (IQR 4–8).

In 18 cases (52.9%), one or more comorbidities that could affect neonatal feeding tolerance were present. In these 18 neonates, enteral feeding was initiated on median postoperative day two (IQR 2–3). After four postoperative days, enteral feeding was initiated for 16 out of 18 neonates (88.9%) (Figure 1A). Enteral feeding was initiated after 18 and 20 days in the remaining two neonates. Full enteral feeding was reached on median postoperative day 6.5 (IQR 5–8.5). On the fifth day after surgery, only five out of 18 neonates (27.8%) had reached full enteral feeding (Figure 1B). Moreover, seven neonates (38.9%) took more than seven days to reach full enteral feeding, with three of them reaching full enteral feeding on day eight and the other four on days 10, 17, 23, and 36. In the neonates requiring 17 and 23 days to reach full enteral feeding, the extended period was more likely due to postoperative complications (a gastric perforation from the nasogastric tube and an esophageal anastomosis leakage after esophageal atresia repair, respectively), rather than the colostomy formation or the comorbidities themselves.

In the 16 neonates without comorbidities that could affect neonatal feeding tolerance, enteral feeding was initiated on median postoperative day two (IQR 1–2), with 100% of them having enteral feeding initiated by postoperative day two (Figure 1A). Full enteral feeding was reached on median postoperative day five (IQR 4–6.75). Five neonates (31.3%) required more than five postoperative days to reach full enteral feeding (Figure 1B): one required six days, two required seven days, one required nine days, and one required 16 days. In these five neonates, feeding could not be advanced more rapidly due to bilious drainage from the nasogastric tube or vomiting, with no other identifiable cause for feeding intolerance besides the colostomy.

During the postoperative course, 11 neonates developed a high-output stoma (defined as >20 cc/kg/day) requiring additional measures (Table 3). In all neonates, the high-output stoma occurred after already reaching full enteral feeding. In two of these 11 cases, enteral feeding was tapered down after two and three days of full enteral feeding. These neonates were back on full enteral feeding after three and six days, respectively.

### 3.3 Central venous catheters and peripherally inserted central catheters

In 17 of 34 neonates (50%), a CVAD was placed: 11 in the 18 patients with comorbidities affecting feeding tolerance and six in the 16 patients without any of these comorbidities. Nine CVADs (52.9%) were placed before colostomy formation or at the time of the procedure, and eight (47.1%) one or more days postoperatively (median postoperative day two, IQR 1–3). In four of these eight neonates—three of whom did not have relevant comorbidities—subsequent anesthesia was necessary to insert the CVAD. The median dwell time for the CVADs was 10 days (IQR 6–13). In the 17 neonates (50%) who did not receive a CVAD, it took five postoperative days to reach full enteral feeding (IQR 4–6).

In seven of 17 neonates with a CVAD (41.2%), there were one or more CVAD-related complications (Table 3). In six neonates, this concerned a suspicion of catheter-related sepsis, which occurred a median of 9.5 days after its placement (IQR 5–13). The suspected catheter-related sepsis was treated with a watchful waiting approach in one neonate, with CVAD removal in three neonates (Clavien-Madadi score IB), and with antibiotics in two (Clavien-Madadi score II). In five cases with suspected catheter-related sepsis, a culture (either blood culture or culture from the removed catheter) was taken, of which only two were positive. In two neonates, fluids extravasated through the CVAD (occurring three and six days after its placement), which required the removal of the CVAD in both. One of these neonates was still dependent on the CVAD and received a new one under general anesthesia (Clavien-Madadi score IIIA).

## 4 Discussion

This study is the first to provide data on postoperative enteral feeding tolerance for neonates undergoing colostomy formation for an ARM specifically. The findings of this study are crucial as they offer new insights into the postoperative course concerning enteral feeding, which can be particularly useful in decision-making, such as whether to place a CVAD perioperatively. This study shows that most neonates who underwent colostomy formation for an ARM take at least five postoperative days to reach full enteral feeding, requiring a prolonged period of IV fluid administration to meet daily fluid targets.

It is recommended to start postoperative enteral feeding as soon as possible (9, 10). In our cohort, enteral feeding was initiated on median postoperative day two, which can be considered relatively late. Moreover, only 47.1% of the cohort had reached full enteral feeding by postoperative day five. Especially neonates who were born prematurely or small for gestational age or had major structural cardiac defects, tracheoesophageal anomalies, abdominal anomalies other than ARM, cerebral anomalies, or syndromic conditions required an extended period to achieve full enteral feeding. However, not all these comorbidities are evident before colostomy formation, making it challenging to identify all affected children before enteral feeding issues arise. Furthermore, this study found that almost one-third of neonates without these comorbidities also required more than five days to achieve full enteral feeding. Therefore, clinical symptoms resulting from the colostomy formation, such as bilious drainage from the nasogastric tube or a persistent distended abdomen, appear to contribute to a prolonged period of inadequate enteral feeding tolerance as well.

If enteral feeding is contraindicated or insufficient, IV fluid supplementation is necessary to meet daily fluid requirements. In our study, 52.9% of neonates required IV fluid supplementation for at least five days after surgery. However, since neonates with an ARM requiring colostomy formation are not allowed enteral feeding in the days before colostomy formation, the total duration of IV fluid requirement was even longer (median of two additional preoperative days). In cases of intermediate- to long-term IV medication or fluid dependency, placement of a CVAD (CVC or PICC) is justified (11, 12). It is recommended to insert a PICC—instead of a peripheral intravenous catheter (PIVC)—in patients who require more than four days of postoperative fluid or IV medication therapy (13). In our study, this applies to 62.5% of the neonates without relevant comorbidities and 83.3% of the neonates with these comorbidities. An important reason for this recommendation is that PIVCs often dislodge, resulting in subcutaneous fluid administration (with the subsequent risk of local tissue damage, infection, compromised blood flow, and nerve injury) and the requirement of repeated needle punctures for replacements (14). Unfortunately, we could not analyze the number of PIVC insertions that could have been prevented by using a CVAD since the number of PIVC insertions per patient is not consistently recorded in our hospital. However, according to the literature, the PIVC failure rate in pediatric patients is 38% and increases to 49% when focusing on neonates specifically (14), emphasizing the need for CVAD placement in all neonates with prolonged IV fluid necessity.

In our study, four neonates—most of whom did not have comorbidities related to feeding tolerance—required an additional procedure under anesthesia for CVAD placement. Anesthesia in pediatric patients, especially in neonates, carries a significant risk of complications. For instance, a study by de Graaff et al. found that critical incidents, such as laryngospasm, hypoxia, hypotension, allergic reactions, and aspiration, occur in 3.4% of pediatric cases, rising to 7.7% in neonates and 10.8% in children with ASA score four or five (15). Given these risks, minimizing the number of anesthetic exposures is important. Inserting a CVAD at the time of colostomy formation reduces the need for additional interventions under general anesthesia. Moreover, it would be beneficial if a CVAD is already in place when an indication for PN arises. It is recommended to initiate PN in critically ill neonates after 48–72 hours and to continue PN in the late acute and recovery phase (16). However, there still is controversy regarding the timing of PN, and it is questionable whether neonates with an ARM with an indication of colostomy formation can be classified as critically ill in this context.

It is important to note that CVADs can lead to complications, such as mechanical problems, infections, phlebitis, and venous thrombosis (11, 12). The overall incidence of CVAD-related complications is reported to be around 30% for PICCs and 16.7% for nontunneled CVCs (17). In our study, the complication rate was 41.2%, which is higher than expected. However, this rate is most likely overestimated since only two of six neonates with a suspected catheter-related infection had a positive blood culture. Additionally, despite one complication with a severity score of IIIA—because of CVAD replacement under anesthesia—none of the CVAD-related complications in our cohort were severe. Nonetheless, it is important to consider the potential risk of CVAD-related complications and to weigh these against the risk of repeated PIVC failure and repeated exposure to general anesthesia.

Many CVAD-related complications may be prevented by implementing several preventative measures, such as using the correct type of catheter, maintaining appropriate hygiene, and performing heparin flushing for intermittent CVAD use (18). Furthermore, minimizing the catheter's dwell time is essential, as the risk of developing CVAD-related sepsis is significantly lower in CVADs with a dwell time of seven days or less compared to more than seven days (19, 20). Our study found that the median dwell time was 10 days. However, it is important to note that there was selection bias in this context. A substantial number of these neonates required CVAD placement during the postoperative course because of pre-existing enteral feeding intolerance, thus preselecting neonates with the most extended IV fluid requirement. Assuming that CVADs will be removed after reaching full enteral feeding, the 17 neonates who did not receive a CVAD would only have needed it for a median of five days, resulting in a lower complication risk. Finally, according to previous literature, CVADs inserted in the operating room are three times less likely to cause CVAD-related infections compared to CVADs inserted in the neonatal intensive care unit (21), which again highlights the benefit of CVAD placement during surgery for neonates undergoing colostomy formation for an ARM.

This study has some limitations. Firstly, it lacks data on the type of enteral feeding (breast milk or formula), whereas previous studies suggest that formula feeding may increase the risk of feeding intolerance (22, 23). However, these studies primarily focused on patients who had not undergone abdominal surgery, raising questions about their relevance to our group. In our institution, breast milk is the preferred type of enteral feeding; however, formula is used when breast milk is unavailable. Unfortunately, the type of enteral feeding is not systematically documented in our institution, preventing us from analyzing the potential relationship between feeding type and postoperative tolerance in our patient group. However, since our study aimed to reflect the time required to achieve full enteral feeding in clinical practice—enabling anticipation and planning—the specific type of feeding is less important in this context, as children will inevitably receive different forms of enteral nutrition based on what is available and their individual circumstances. Secondly, we were unable to evaluate how inadequate nutrition may have contributed to postoperative complications, such as wound infections. This limitation arises from two factors: first, only a few patients experienced such complications (four cases of wound infections), and second, we could not quantify malnourishment through laboratory values, as routine testing was not performed. However, it is reasonable to assume that children with poor nutritional status are more vulnerable to postoperative complications. As our results clearly indicate that many neonates with ARM tolerate little to no enteral feeding for prolonged periods after colostomy formation, our findings underscore the importance of addressing and anticipating these challenges related to feeding tolerance. Finally, it is important to interpret the findings of this study with caution, as our analysis is retrospective and exploratory in nature. Future prospective multicenter studies are warranted to further validate our observations and recommendations.

The study's primary strength is that—to our knowledge—it is the first to investigate the postoperative feeding tolerance and subsequent need for a CVAD after colostomy formation in neonates with ARM. Moreover, even though ARM is a rare condition, we successfully gathered a relatively large cohort for this analysis. These factors enhance the study's value, providing insights that can directly inform and improve clinical practices in managing these patients.

## 5 Conclusions

This study underscores the prolonged period required for achieving full enteral feeding in neonates undergoing colostomy formation for ARMs. Among the neonates with comorbidities affecting feeding tolerance, over 72% required more than five days to reach full enteral feeding. To bridge this prolonged period of inadequate enteral feeding and to reduce the significant risks associated with repeated anesthesia, we advocate for the routine placement of a CVAD (either PICC or CVC, depending on hospital preference) during surgery for neonates with known comorbidities affecting feeding tolerance. Although less common, more than 30% of neonates without these comorbidities still required more than five days to achieve full enteral feeding. Therefore, for neonates without known comorbidities affecting feeding tolerance, perioperative CVAD placement should be considered, balancing the risks of CVAD placement against the risks of repeated PIVC insertion and multiple exposures to general anesthetics. Appropriate measures should be taken to minimize CVAD-related complications, including timely removal of the CVAD after achieving full enteral feeding.

## Data Availability

The raw data supporting the conclusions of this article will be made available by the authors, without undue reservation.
